# Rare constellation of abdominal vascular injuries in blunt trauma: Left gastric artery pseudoaneurysms and dissection

**DOI:** 10.1016/j.ijscr.2019.05.028

**Published:** 2019-05-24

**Authors:** Karan D’Souza, Michael Sean Bleszynski, Harvey George Hawes

**Affiliations:** a Division of General Surgery, Department of Surgery, University of British Columbia, 855 West 12th Avenue, Vancouver, BC, V5Z 1M9, Canada; b Division of General Surgery, Department of Surgery, University of British Columbia, Vancouver, BC, V5Z 1M9, Canada; c Department of Trauma, Vancouver General Hospital, Vancouver, BC, V5Z 1M9, Canada

**Keywords:** Case report, Left gastric artery, Pseudoaneurysm, Dissection, Blunt trauma

## Abstract

**Introduction:**

Abdominal visceral artery pseudoaneurysms and dissections in blunt trauma are uncommon, however, solitary vascular injury of the left gastric artery and development of multifocal dissections are extremely rare with only 5 reported cases in the literature.

**Presentation of case:**

A 79-year-old male presents two days after a motor boating incident in profound hemorrhagic shock. Subsequent CT imaging revealed moderate hemoperitoneum, two pseudoaneurysms of the left gastric artery measuring 6 mm and 9 mm, as well as attenuation of the artery in keeping with a focal dissection and intramural thrombus, and no active extravasation. After stabilization with blood product resuscitation, he was managed conservatively with administration of ASA and close monitoring.

**Conclusion:**

Abdominal visceral artery vascular injuries in blunt trauma can be managed safely with close monitoring, antithrombotic agent, and medical co-morbidity optimization. Instability or worsening/ persistent symptoms should be considered for endovascular treatment or surgical ligation Further research and reporting of management approaches are required.

## Highlights

• Traumatic visceral artery pseudoaneurysms and dissections are typically secondary to penetrating trauma involving splenic or hepatic vessels.
• Solitary left gastric pseudoaneurysm and dissection in blunt trauma are rare, and best recognized using multi-detector multiphase CTA.
• Pseudoaneurysms measuring less than 2.0 cm without symptoms of persistent abdominal pain or peritoneal signs can be managed conservatively.
• Conservative management involves close observation, treatment with antithrombotic agents, and optimization of medical comorbidities.

## 1 Introduction

Abdominal visceral artery vascular aneurysms include both true aneurysms, which have all three layers of the arterial wall involved, and false aneurysms, also known as pseudoaneurysms, that lack complete involvement of all arterial wall layers. Distribution of aneurysms within the abdomen mainly occur in the splenic artery (60%) and hepatic artery (20%), with only 4% of aneurysms being found in the gastric and gastroepiploic arteries [[1], [2], [3]]. We detail a patient who presented to the emergency department in a delayed fashion in hemorrhagic shock secondary to abdominal vascular injuries after motor boat incident. Pseudoaneurysm of the left gastric artery and subsequent dissection was detected on computed tomography (CT) and was managed conservatively with close observation in a monitored setting and antithrombotic agents, by multidisciplinary team.

## 2 Case report

A 79-year-old Caucasian male, with a past medical history of atrial fibrillation on warfarin and metoprolol, and coronary artery disease on atorvastatin with previous coronary artery bypass grafting and placement of a dual-function pacemaker/ implantable cardioverter defibrillator (ICD), was on a motor boat in a remote location. The patient’s boat went over a wake of a larger boat passing by. He bounced off his seat in a vertical direction and subsequently landed on his tailbone. After the high impact fall, he complained of both immediate lower back and diffuse abdominal pain but did not seek out urgent medical help.

Two days after the initial incident, he started to become pale and diaphoretic; additionally, his ICD delivered three shocks over a 30-min period. He presented via ambulance service to a local community hospital in hemorrhagic shock with a blood pressure of 63/22 and heart rate of 118 beats/min. A primary survey was pertinently positive for hemodynamic instability and diffuse abdominal and lower thoracic spine tenderness.

He was resuscitated with 1 L of normal saline leading to an improvement of his pressure to 106/88. Initial laboratory investigations included a hemoglobin of 95 g/L, lactate of 6.1 mmol/L, creatinine of 129, and a supratherapeutic INR of 8.8. An initial non-contrast CT abdomen and pelvis showed moderate hemoperitoneum with sentinel clot in the left upper quadrant and pericolic gutter, as well as the area adjacent to the posterior wall of the stomach. An additional finding of a severely comminuted, minimally displaced burst fracture of the T10 vertebral body was noted (Fig. 3). Further interventions included INR reversal with 3 mg of Vitamin K and 3000 units of prothrombin complex concentrate, and administration of 2 units of packed red blood cells and 2 L of normal saline. Based on clinical severity, the patient was transferred to the trauma service at a tertiary-care Level 1 trauma center.

**Fig. 1:**
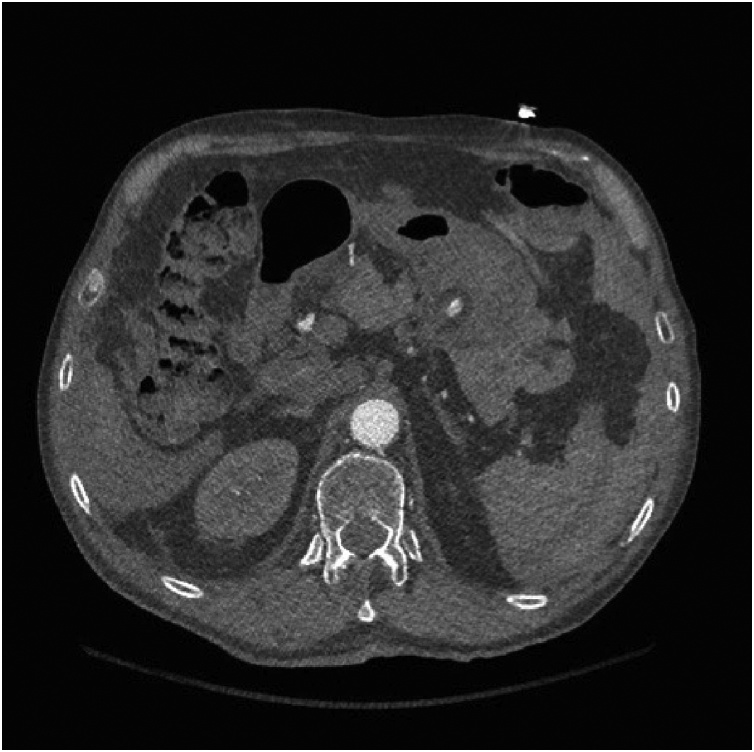
CT Angiogram in the arterial phase demonstrating a 9 mm focal area of increased attenuation along the lesser curvature of the stomach representing a small pseudoaneurysm of the left gastric artery, with no active extravasation appreciated.

**Fig. 2:**
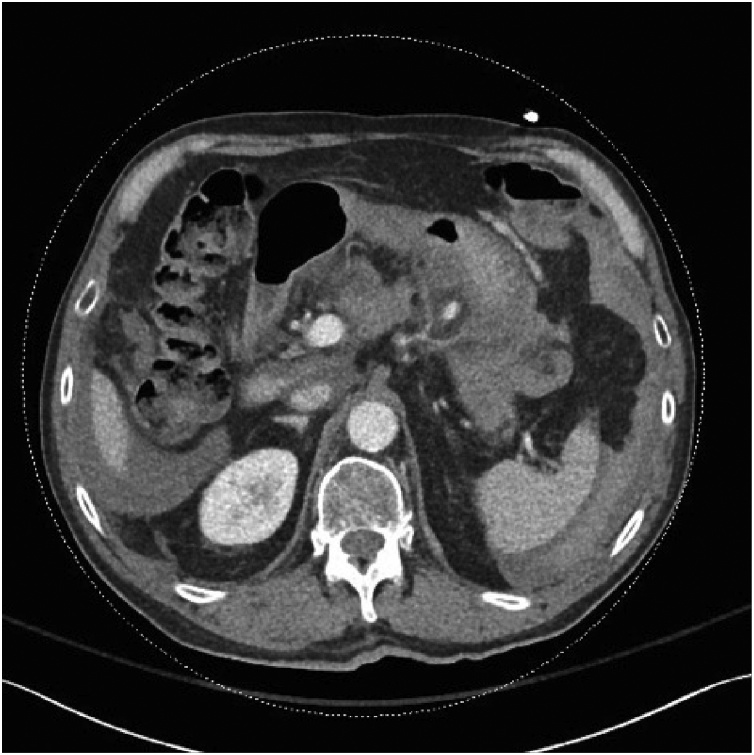
CT Angiogram in the portal venous phase demonstrating stable pseudoaneurysms of the left gastric artery and interval development of left gastric artery attenuation in keeping with a focal dissection and intramural thrombus.

Primary survey revealed a protected airway, spontaneous and bilateral air entry, and hemodynamic stability with a blood pressure of 100/60 and a heart rate of 88 beats/min. His abdomen continued to be mildly distended and tender without peritoneal signs, however the patient reported it had improved since his original presentation to the local hospital. Repeat laboratory investigations revealed a stable hemoglobin of 94 g/L, and correction of his INR to 1.2. Given his stable condition, he underwent a CT RIPIT (Rapid Imaging Protocol in Trauma) [4] and CT angiogram (CTA) of the abdomen and pelvis. His imaging revealed pseudoaneurysms of the left gastric artery measuring up to 6 mm with another 9 mm rounded area of increased attenuation along the lesser curve of the stomach (Fig. 1, Fig. 2). No extravasation was seen. Decision was made to monitor the patient closely with serial abdominal exams and repeat imaging in 72 h, or sooner if the patient exhibited any signs of deterioration.

**Fig. 3:**
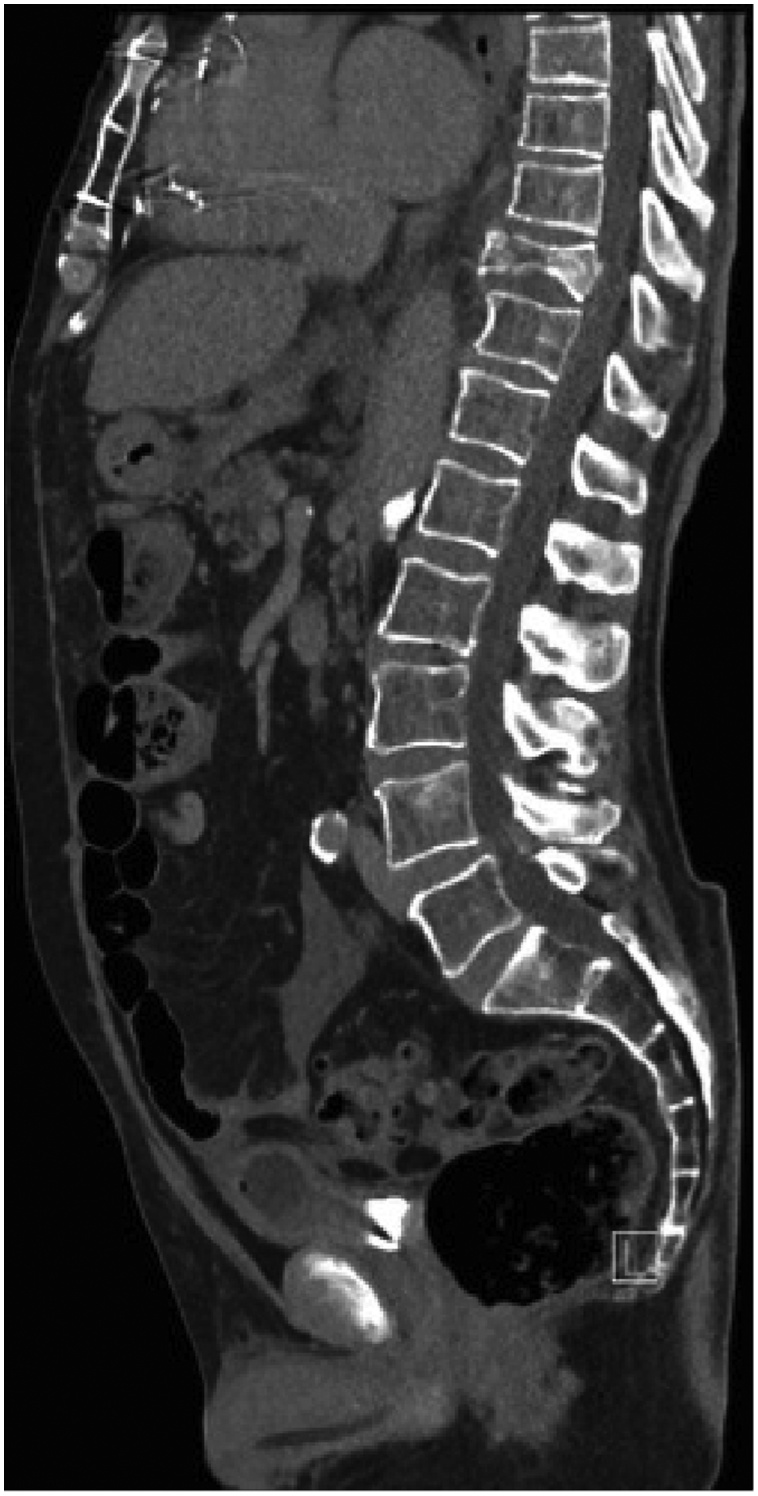
In addition to the vascular injuries, the patient also had a severely comminuted, minimally displaced burst fracture of the T10 vertebral body resulting in 63% height loss. Retropulsion of the posterior inferior endplate of 2 mm and anterior displacement of the anterior vertebral body of 7 mm are also noted.

Over the next 72 h, the patient’s vital signs and abdominal exams, improved and his hematological profile remained stable. Given the patient’s CHADS score of 2, it was decided to hold therapeutic anticoagulation until the patient’s bleeding risk decreased, however deep venous thrombosis prophylaxis was initiated. A repeat CTA was performed to follow the evolution of the two pseudoaneurysms. The imaging study revealed unchanged pseudoaneurysms but noted the distal aspect of the left gastric artery was attenuated in keeping with a focal dissection and intramural thrombus. Secondary to the dissection, the patient was started on 81 mg of aspirin daily. Over the next few days the patient continued to improve clinically, and was discharged home. The patient lived outside of the local area, and arrangements for close follow up were made.

## 3 Discussion

We report a case of left gastric pseudoaneurysms with development of multifocal dissections secondary to a blunt traumatic injury. After initial resuscitation, the patient was hemodynamically stable without peritoneal signs, as such we elected to manage the patient conservatively with medical therapy. Since his discharge, he has remained stable and asymptomatic.

Left gastric pseudoaneurysms are uncommon, and in the context of an associated arterial dissection, where a tear forms along the intima and forms a false lumen, is exceedingly rare. Additionally, in traumatic visceral artery pseudoaneurysm cases the etiology is more frequently observed in penetrating trauma, hence our case is a unique addition to the current literature [5].

Recognizing visceral abdominal vascular injuries can be accomplished using a variety of imaging modalities. Ultrasound provides a method with no radiation exposure and is low-cost and non-invasive. However, it is most useful for larger vessels, and is limited by technician expertise and prevalence of bowel gas that obscures visualization of mesenteric and retroperitoneal vessels. Multi-detector multiphase CT is the most frequently used modality, especially in trauma, as it provides further characterization of the vascular abnormality and illustrates approaches for endovascular management [1].

Given the rare presentation of traumatic left gastric vascular injury, there are no clear guidelines regarding treatment recommendations. Management has been based on expert opinion and published case reports (Table 1) where endovascular stenting and coiling are the mainstays of treatment [[6], [7], [8], [9], [10]]. Nonetheless, management should be individualized to the particular case and clinical presentation.

**Table 1:** Previously reported cases of left gastric artery injury in the setting of blunt trauma.

Study	Age/ Sex	Mechanism	Clinical Findings	Imaging Findings	Assoc. Injuries	Treatment
Yunoki et al. [4]	58; M	Minor road traffic accident involving semi-trailers	Delayed (>12 h) worsening epigastric pain; subsequent hemodynamic instability with BP 86/62, and 16 point drop in hemoglobin	**CT**: Presence of intraperitoneal fluid collections in the superior and inferior recesses of lesser sac, perihepatic and perisplenic space.	None	Disrupted branch of left gastric artery along the lesser curvature identified and ligated during laparotomy
Valera et al. [5]	43; M	Head-on MVC; unrestrained	Hemodynamically stable; Diffuse abdo. tenderness without peritonitis; Persistent tachycardia, and metabolic acidosis	**CT/CTA**: Minimal free fluid; Hematoma on inferior aspect of left hepatic lobe; left gastric artery pseudoaneurysm with acute extravasation along lesser curvature of stomach	Left anterior 7 and 8 rib fractures; L5 right transverse process fracture; left acetabular fracture	Embolization with microcoils followed by a single pledget of Gelfoam
Matthew et al. [6]	24; M	Blunt abdo. trauma; delayed presentation (10 days)	Massive bleeding through abdominal drain after distal pancreatectomy and splenectomy secondary to near total transection of the pancreas.	**CTA:** Selective visceral angiogram depicted a 2.5 cm pseudoaneurysm arising from terminal end of descending left gastric artery	Post-operative thrombocytosis and generalized tonic clonic seizure	left gastric artery trunk embolized with coils and poly vinyl acrylate particles
Allorto et al. [7]	19 mon; M	Non-accidental blunt trauma	Hemodynamically stable with anemia; Abdominal pain, distention and vomiting; Palpable upper abdo. mass.	**MRI**: Lesion between left hepatic lobe and stomach. **CTA**: Small pseudoaneurysm identified arising from one of the left gastric branches.	Previous healed clavicle fracture	Pseudoaneurysm and segment of the vessel proximal and distal to aneurysm were occluded with four detachable coils
Nissim et al. [8]	25; M	Head-on MVC; restrained	Hemodynamically unstable with HR 110-120 and MAP 67; Epigastric tenderness and “seat belt sign”	**CT**: Large (4.8 × 2.9 × 5.8 cm) hematoma in lesser sac of abdomen and active blush arising from the left gastric artery.	Extremity fractures	The left gastric branch vessel was embolized with a two detachable coils; Proximal artery coiled with 2 detachable coils to stasis.

*Acronyms –* M: male, Mon: months, MVC: motor vehicle collision, BP: blood pressure, Abdo: abdomen, HR: heart rate, MAP: mean arterial pressure, CT: computer tomography, CTA: computer tomography angiography, MRI: magnetic resonance imaging.

The management of traumatic and spontaneous visceral artery dissections is dependent on patient symptomology and clinical status [[11], [12], [13], [14]]. Advancement in endovascular techniques have led to many surgeons electing to use stenting as an initial approach for patients with persistent abdominal pain, peritoneal signs, aneurysms greater than 2.0 cm, or presence of bowel ischemia [12,13]. Open surgical intervention with vessel over-sew or primary repair continues to be an option for definitive management, but is usually reserved for patients who have failed endovascular management or therapeutic anti-thrombotic therapy, as it carries higher risk of morbidity for patients [11,13]. Therefore, endovascular and open surgical approaches are recommended in patients who are hemodynamically unstable, have evidence of organ necrosis secondary to dissection, or when vessel rupture is present [13].

Recently, systematic reviews show that conservative management with close monitoring, antiplatelet agents or therapeutic anticoagulation are safe, and do not negatively impact patient outcome [[11], [12], [13], [14]]. Antiplatelet agents or therapeutic anticoagulation are used to stabilize the vascular wall injury and prevent thrombotic occlusion. Medical therapy does require close monitoring for development of ischemia and signs of progression of vascular injury or rupture. Additionally, they require effective management of their co-morbidities, especially hypertension. For this patient, given the delayed presentation and no clear source for the hemoperitoneum and no active extravasation on the initial CT angiogram (CTA) scan, a decision was made to repeat a CTA and monitor the pseudoaneurysm. We also elected to monitor and perform serial abdominal exams without initiation of anti-coagulation. We initiated 81 mg daily aspirin once the repeat CTA showed no evolution of the vascular injuries and no further intra-abdominal pathology.

## 4 Conclusion

In summary, blunt trauma causing two left gastric pseudoaneurysms with multifocal dissections can be managed conservatively with administration of ASA. High fidelity imaging plays an important role in management of a stable patient with blunt traumatic visceral abdominal vascular injuries. However, instability or worsening symptoms should be considered for immediate endovascular treatment or surgical ligation.

## Conflicts of interest

None.

## Funding

None.

## Ethical approval

Ethics Approval was not required for this manuscript.

## Consent

The patient gave his verbal and written informed consent to be included in this report as well as for publication of anonymized data and images. This can be provided to the editors, if required.

## Author contribution

*Case Supervision and Study Conception*: D’Souza, Bleszynski and Hawes; *Literature Search:* D’Souza and Bleszynski; *Data Collection*: D’Souza; *Data Analysis:* D’Souza, Bleszynski and Hawes; *Writing:* D’Souza and Bleszynski; *Critical revision:* D’Souza, Bleszynski and Hawes; *Approval of Final Manuscript:* D’Souza, Bleszynski and Hawes.

## Registration of research studies

None. As the submitted work is a case report, there was no study involved.

## Guarantor

Dr. Karan D’Souza, Dr. Michael Bleszynski, and Dr. Harvey Hawes takes responsibility for the integrity of the work and the accuracy of the manuscript.

## Provenance and peer review

Not commissioned, externally peer-reviewed.
